# Urine π-Glutathion S-transferase but not Tamm-Horsfall protein correlates with Carotid artery intima media thickness in childhood type1 diabetes

**DOI:** 10.1186/1471-2261-14-39

**Published:** 2014-03-26

**Authors:** Peter Holmquist, Petru Liuba

**Affiliations:** 1 Division of Endocrinology, Department of Paediatrics, Lund University Hospita, S-22185 Lund, Sweden; 2 Pediatric Heart Center, Lund University Hospital, S-22185 Lund, Sweden

**Keywords:** π-GST, Vascular endothelial function, Intima media thickness, Type1 diabetes

## Abstract

**Background:**

Renal disease remains a serious threat in patients with insulin-dependent (type1) diabetes. Hence its detection early in the life of patients with type1 diabetes is crucial. Several lines of evidence suggest similar mechanisms for the development of both renal and arterial disease. We sought to investigate in young patients with type1 diabetes whether π-Glutathione S-transferase to creatinine (π-GST:crea) and Tamm-Horsfall protein to creatinine (THP:crea) ratios, markers of distal tubular renal function, relate to subclinical markers of arterial disease, which appear to onset early and develop rapidly in type1 diabetes.

**Methods:**

Seventy-one children and adolescents (median age and diabetes duration 14 and 6 years, respectively) with type1 diabetes for at least 6 months were assessed for timed urine levels of π-GST, THP, HbA1c, albumin, and plasma C-reactive protein (CRP). Carotid artery intima-media thickness (IMT), brachial artery flow-mediated dilatation (FMD), and cutaneous microvascular function were assessed by high-resolution ultrasound and laser Doppler, respectively.

**Results:**

Two patients had microalbuminuria (> 20 μg/min), and were therefore removed from the study population. π-GST:crea ratio and THP:crea showed no relationship to the demographic, diabetes, or inflammatory indices. Lower π-GST:crea ratio was associated with greater IMT (p = 0.01, r = −0.29), particularly in female patients (p = 0.004, r = −0.49). The association of π-GST:crea ratio with IMT was stronger in patients with passive smoke exposure (p = 0.002, r = −0.43). Among post-pubertal patients, lower π-GST:crea ratio was also associated with lower microvascular response to Ach (acetylcholine; p = 0.03, r = 0.49).

**Conclusions:**

In young patients with type1 diabetes, proximal tubular dysfunction as suggested by lower levels of π-GST:crea ratio seems to be paralleled by changes in arterial structure and microvascular function.

## Background

Functional and structural arterial abnormalities occur early in the life of patients with type1 diabetes [1-3]. Thickening of the arterial wall and functional disturbances in the arterial endothelium, which are more prevalent in type 1 diabetes particularly when other risk factors such as smoke exposure are present [1], may be detected without any clear relation to the degree of glycemic control [4]. Although subtle, these changes may be detected non-invasively via high-resolution ultrasound and are seemingly at least in part responsible for the increased prevalence of cardiovascular disease in adult life [2].

In the kidney, microvascular dysfunction is thought to contribute to tubular injury [3]. Albuminuria is still the accepted standard in the diagnosis of nephropathy, and in some but not all studies was found to be associated with vascular changes [5,6]. Microalbuminuria, however, has a low sensitivity and specificity especially in young patients [7].

Novel urine biomarkers specific for different parts of the kidney have been tested for early diagnosis of nephropathy [7,8]. One of these, π-GST, is localized in the cytoplasm in both podocytes and parietal epithelial cells of Bowmans capsule and mostly in the distal convoluted and medullary tubules and collecting ducts [9]. Recent study showed that π-GST is also expressed in aortic endothelial cells [10], and may protect vascular endothelium against noxious stimuli. In adult patients, it has been found to be a predictor of kidney injury with increasing albuminuria [7]. In a male cohort of pediatric patients with type1 diabetes, π-GST:crea ratio was decreased [11], but increase has been reported after cardiac surgery [12].

Another putative marker of tubular dysfunction, Tamm-Horsfall protein (THP), is located in the thick ascending limb of the loops of Henle and the distal tubular cells [13], Its urine excretion is largely regulated by sodium intake [14]. Abnormal urine excretion of THP is found already at diagnosis of type1 diabetes in children [8]. An association between cardiovascular mortality and decreased levels of urine THP in adult type1 patients has been reported [15].

Our aim was to assess whether urine levels of π-GST:crea ratio and THP:crea are associated in young patients with type 1 diabetes with changes in vascular structure and function.

### Subjects

Of the 184 children with type1 diabetes (duration > 6 months) enrolled in a previous study on urine excretion of GST enzymes [16], 71 were included in another study carried out within the same timeframe investigating vascular (carotid artery intima media thickness (IMT), and skin microvascular endothelial function) and inflammatory (C-reactive protein (CRP)) parameters. Exposure to environmental tobacco smoke (ETS) was assessed via questionnaire [1], and defined as occasional or regular cigarette smoking in the presence of study participants in or outside home (e.g. private or public transportation, in or around school, playground, other public places). Patients were divided into three groups: 1 = no exposure during the past year; 2 = occasional exposure, i.e. presence in a smoky environment less than once a week; 3 = weekly to daily exposure. In addition the average number of cigarettes smoked per day in or around home by patient’s cohabitants was assessed as well as the number of household smokers.

Our patients are routinely treated with insulin Glargin once daily or insulin Detemir twice daily and direct-acting insulin Aspart with meals. In this cohort, nearly 15% of the diabetic children used an insulin pump with direct-acting insulin. HbA1c and blood pressure were measured four times a year upon follow-up visits.

Exclusion criteria were age below 6 years, familial hypercholesterolemia, active smoking, systemic hypertension, and albuminuria (urine albumin > 20 μg/min). The ethical committee for human research at the Lund University Hospital approved the study (571/2004). Written and oral consent was obtained from all participants and or parents.

## Methods

### Urine and blood analyses

The method for urine π-GST analysis is described elsewhere [11]. Timed-over-night urine was collected at home. A specimen of urine for GST was spared with the addition of a preservative provided by the manufacturer (Biotrin International Ltd, Dublin, Ireland). Seventy-one π-GST urine tests were analyzed with a commercially available solid phase sandwich, immunosorbent assay from Biotrin International Ltd, Dublin, Ireland. HbA_1c_ was measured by a high performance linked liquid chromatography (HPLC, Auto-A, Tosoh) with a normal value of 4–5.3%. U-creatinine was measured by an enzymatic calorimetric method (Hitachi Modular-P) with detection limit 0.03 mmol/L (range 0.03-53 mmol/L). Urine Tamm-Horsfall protein [17] and albumin [18] were measured by ELISA as previously described. Plasma high sensitive C reactive protein (CRP) was measured by enzyme-linked immunoassay using polyclonal antibodies (DACO Diagnostics, Glostrup, Denmark). Plasma cystatin C was measured by an automated particle enhanced immunoturbidimetric method with normal range of 0.55-1.15 mg/L [19]. Plasma creatinine was analysed by a creatininase enzyme-based analyse system (Hitachi Modular-P) with detection limit of 2.7 μmol/L and a normal value between 5–15 years 25–68, male 60–105 and female 45–90 μmol/L. All children had their height and weight measured. Blood pressure was taken in supine position after 5-minute rest with an arm cuff covering two third of the right upper arm.

### Assessment of carotid artery intima-media thickness

Longitudinal scans in bidimensional mode of the 1-cm long distal end of the left common carotid artery were imaged so that the lumen-intima and intima-media interfaces were clearly distinguishable. All scans corresponded to the R-wave on the ECG. Four to six scans from each individual were recorded on videotape for off line analyses of the carotid artery compliance, stiffness index and intima media thickness IMT). The mean carotid IMT of four measurements along a 1-cm segment was calculated from each scan. Mean IMT values obtained from all scans from the same subject were averaged, and the resulted mean IMT was used for statistical analyses. All scans were taken by a single sonographist, and the analyses were performed blind to the patients’ characteristics. The intraobserver variability coefficient was less than 5%.

### Cutaneous microvascular function by laser doppler with iontophoresis

Cutaneous blood flow responses to endothelium-dependent and independent agonists were assessed by using a laser Doppler multifiber probe (481–1; Perimed AB, Sweden) during transdermal iontophoresis of acetylcholine (Ach) and sodium nitroprusside (SNP), respectively, on the volar side of the forearm. The nondominant upper extremity was chosen in all patients. Anodal iontophoresis was used for Ach, whereas SNP was delivered via cathodal iontophoresis. The current was set at 100 μA for 20 s for both drugs, based on previous work [20]. Five consecutive doses were applied for both drugs to generate dose–response curves. Baseline perfusion and changes in response to Ach were expressed as area under the curve. The intraobserver variability for skin responses to acetylcholine was 10.4%.

### Statistics

Results are given as median and range for variables with skewed distribution (see below), and as mean and standard deviations for those variables with Gaussian distribution. Spearman’s correlation coefficient and analysis of variance (ANOVA) followed when applicable (i.e. significant p value) by Bonferroni posthoc test were used to assess the differences between the groups. When variable’s distribution was skewed, log-transformation was used before being entered into the analysis. For statistical purpose (calculation and graphing), patients with π-GST below the detection limit were assigned a value of 0.05 ng/ml. Adjustment for co-variables was done with ANCOVA. Significance was accepted when p ≤0.05. All analyses were performed using the Stat View for Windows as statistical package (USA).

### Calculations

Urinary excretion rates were calculated in relation to 1.73 m^2^ body surface area (= Weight^0.425^ × Height^0.725^ × 71.84/100) to adjust for size and gender. The ratio between the urine component and urine creatinine was used to correct for failures in timed collections. Cystatin C clearance was used to estimate glomerular filtration rate (GFR; ml/min/1.73 m^2^ body surface area) = 84.69 * cystatin C (mg/L) and * 1.384 for children < 14 years [21]. Urine π-GST:crea ratio below 0.03 or above 1.88 μg/mmol the lowest and the highest, respectively, in the control material, was considered abnormal [11].

## Results

π-GST:crea ratio was available in 71 patients. Data from two patients were discarded due to microalbuminuria (urine albumin > 20 μg/min). Demographic, diabetes, inflammatory, metabolic and renal function indexes in the remaining 69 patients are given in Table 1.

**Table 1:** Descriptive data of the studied cohort

	**Mean**	**Std. Dev**	**Count**	**Minimum**	**Maximum**	**Skewness**	**Median**	**IQR**
Age (years)	14.7	3.5	69	7.0	20.0	-.3	14.0	5.0
BMI (kg/m^2^)	20.9	3.2	69	14.9	28.6	.2	20.8	4.9
Diabetes Duration (years)	6.9	4.2	69	.5	18.0	.4	6.0	6.0
HbA1c (%)	6.9	1.3	69	3.9	11.2	.5	67	1.6
Systolic BP (mm Hg)	112.8	10.9	69	81.0	139.0	-.1	70.0	8.0
Diastolic BP (mm Hg)	70.6	6.7	69	53.0	89.0	-.1	70.0	8.0
AER (ug/min)	2.9	2.3	69	.3	11.8	2.0	2.3	2.3
Urine THP: creatinine ratio	.9	.9	69	.0	4.4	2.0	.6	.8
Urine π-GST: creatinine ratio	.5	.9	68	.0	6.0	4.4	.3	.6
Cystatin C (mg/L)	.8	.1	67	.4	1.1	-.6	.8	.2
GFR (ml/min)	145.1	47.6	67	79.0	373.0	2.1	141.0	43.8
CRP (mg/L)	1.8	3.2	68	.1	13.3	2.3	.5	1.4

IQR: interquartile range; BMI: body mass index; HbA1c: glycosylated hemoglobin; BP: blood pressure; AER: albumin excretion rate; THP: Tamm-Horsfall protein; π-GST: π-Glutathione S-transferase to creatinine; GFR: glomerular filtration rate; CRP: C-reactive protein.

### Urine albumin excretion

With the exception of the two aforementioned patients, urine albumin was within normal range. Neither plasma cystatin C nor GFR (cystatin C clearance) showed signs of overt renal disease (Table 1). Albumin excretion rate (AER) rose with increasing age (p = 0.008, r = 0.31) and diabetes duration (p = 0.017, r = 0.29), but remained within normal range in all patients (Table 1). Neither π-GST:crea ratio nor THP:crea correlated with AER (p > 0.2). HbA1c showed no association with AER (p = 0.6), whereas patients without ETS (group 1) tended to have lower AER than those with regular ETS (group 3; p = 0.05, adjusted for age via ANCOVA).

### Urine THP to creatinine ratio

THP:crea ratio, a distal tubular marker of nephropathy, showed no relationship with any of the studied kidney and vascular markers (p > 0.2). Also, there was no correlation between THP:crea and π-GST:crea ratios (p = 0.4).

### Urine π-GST to creatinine ratio

Among patients, π-GST:crea ratio was lower in males than in females (p < 0.01). There was a weak correlation between π-GST:crea ratio and GFR (p = 0.02, r = 0.3). Neither the degree of glycemic control, as expressed by HbA1c, nor the age or diabetes duration, correlated with π-GST:crea ratio (p > 0.4). CRP showed a weak correlation with π-GST:crea ratio (p = 0.03, r = 0.26).

**Figure 1:**
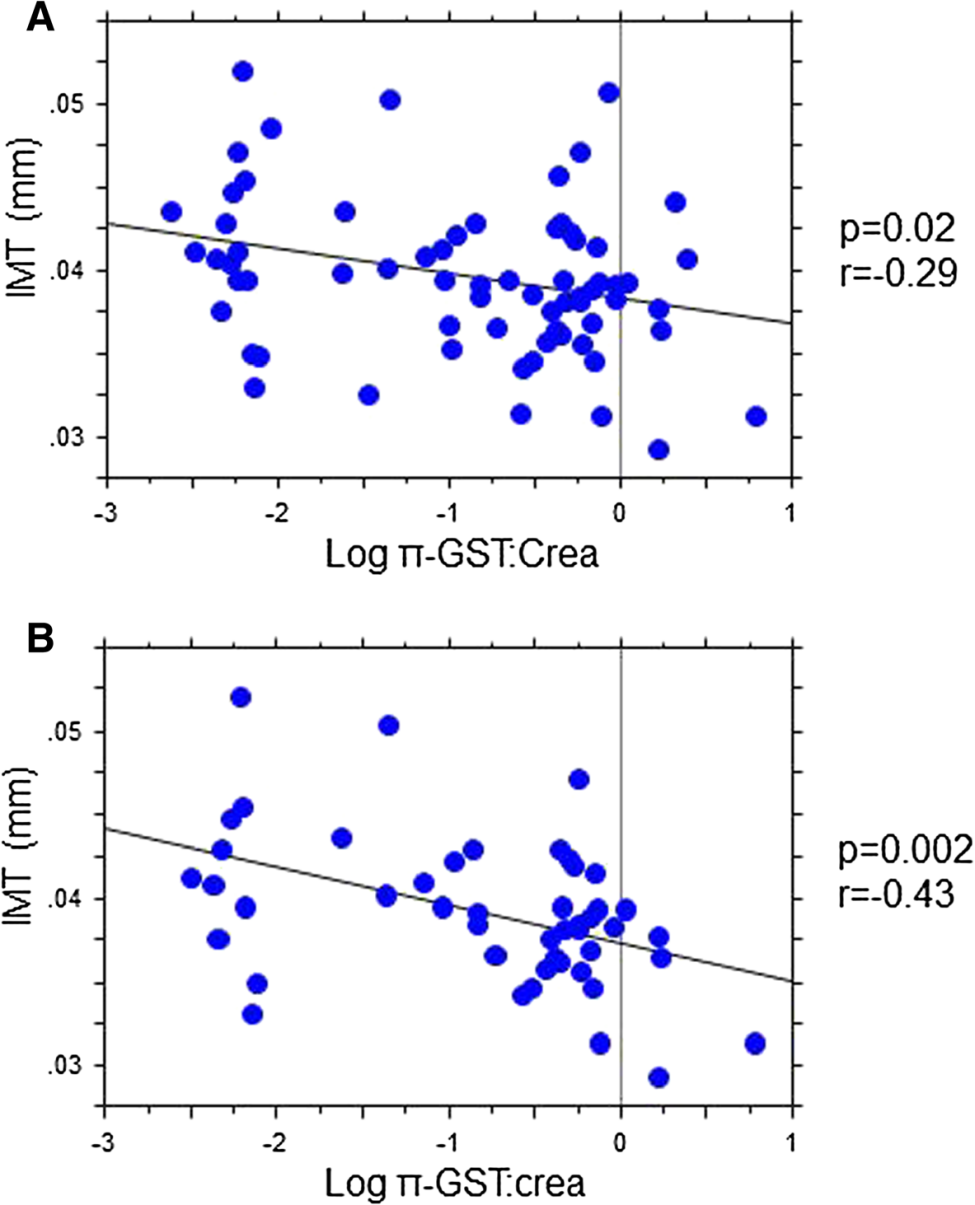
**Relationship of urine π-GST:crea ratio (log-transformed) with carotid artery intima-media thickness (IMT).** Panel **A**: in the whole cohort of patients; Panel **B**: in patients with exposure to tobacco smoke.

**Figure 2:**
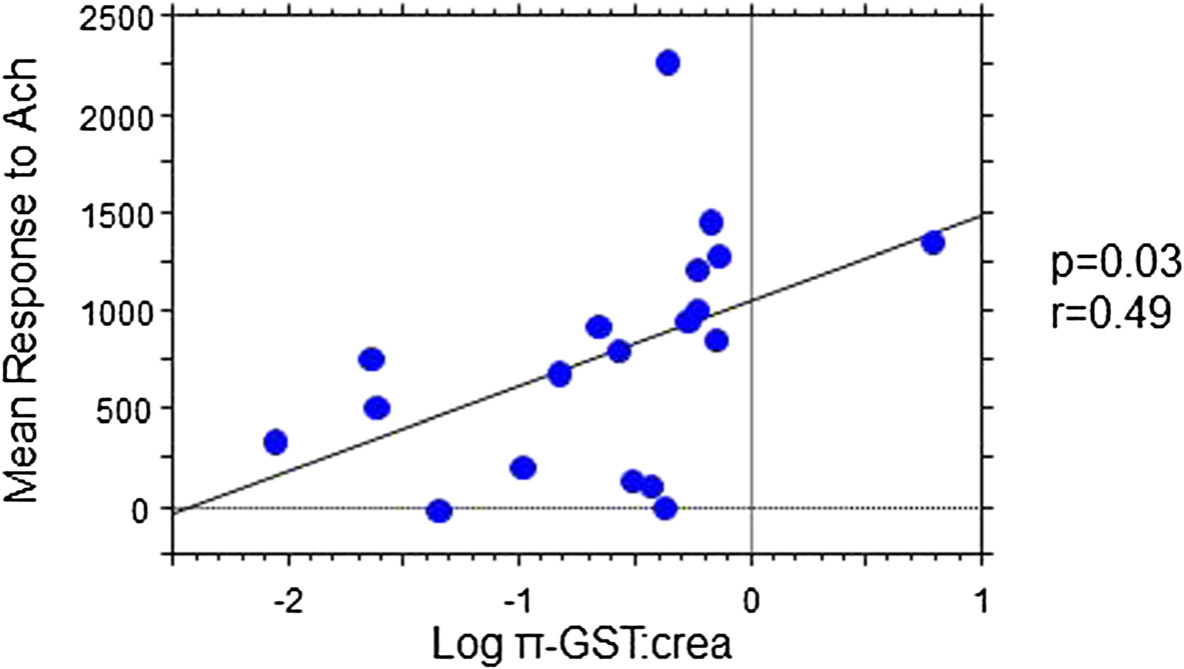
**Relationship of urine π-GST:crea ratio (log-transformed) with mean cutaneous response to acetylcholine (Ach) among postpubertal patients (age ≥14 years).**

Lower π-GST:crea ratio was associated with greater IMT (p = 0.01, r = −0.29; Figure 1/Panel A), particularly in female patients (p = 0.004, r = −0.49). The association of π-GST:crea ratio with IMT became stronger in patients with ETS (p = 0.002, r = −0.43; Figure 1/Panel B). Among post-pubertal patients (>14 years of age), lower π-GST:crea ratio was associated with lower microvascular response to acetylcholine (Ach; p = 0.03, r = 0.49; Figure 2). The responses to SNP showed no correlation with π-GST:crea ratio levels (p > 0.5).

## Discussion

Using a cross-sectional design, we observed in a relatively small cohort of young patients with type1 diabetes several associations between lower urine levels of π-GST:crea ratio and adverse changes in carotid artery structure and cutaneous microvascular function. Female gender and exposure to tobacco smoke appear to strengthen this association. This is in keeping with some previous studies showing that girls with type1 diabetes are more prone to atherosclerosis development [22], and that exposure to tobacco smoke further increases cardiovascular risk among type1 diabetes patients [23].

The development of renal disease in type1 diabetes is often subtle especially in younger patients. The test recommended by the American Diabetes Association for diagnosing and monitoring diabetes nephropathy is the urinary albumin:creatinine ratio; however, this test performs poorly, with relatively low sensitivity and low specificity (7).

In view of the previous studies suggesting that vascular and kidney disease in type1 diabetes progress hand in hand [24,25], the association of π-GST, a novel marker of kidney disease, with increased carotid artery intima-media thickness and cutaneous microvascular dysfunction in the present study is perhaps not surprising. These two adverse vascular changes have been shown to develop early after the onset of type1 diabetes [26]. Abnormalities in peripheral arterial function and structure have been documented in both pediatric and adult cohorts with renal disease [3,27], suggesting a complex “cross-talk” between the vascular system and the kidney [28].

With the exception of this study, to the best of our knowledge, there are no further reports on π-GST:crea ratio in a pediatric population. In adult diabetic patients, urinary π-GST:crea ratio was found to rise with increasing albuminuria [7]. A similar trend was observed in adult patients with overt renal disease [13]. The cohort included in the present study is much younger and free of albuminuria, and was earlier found to have lower levels of π-GST:crea ratio than in healthy controls [11]. Whether age, diabetes duration, and/or stage of kidney injury could influence the urine levels of π-GST remains to be assessed in future studies. Maybe, since the origin of π-GST is renal, there is a bimodal response of kidney π-GST:crea ratio to diabetic kidney injury with an early decrease followed by increase as microalbuminuria occurs.

We noted a weak correlation of π-GST:crea ratio with the inflammatory marker CRP. Smoke exposure, diabetes duration, and poor diabetes control (HbA1c), may all be involved in the onset and progression of endothelial damage in type1 diabetes, with release of inflammatory mediators [4,29]. Intuitively sustained injury to the vessel wall could provide a potential substrate for injury to renal tubules [30]. This could in turn, over time, lead to increase in π-GST excretion. Alternatively, these two processes could be unrelated to each other, although, at least based on our findings suggesting some association between π-GST and IMT, a common mechanism seemingly related to the diabetes milieu (e.g., inflammation, oxidative stress, and hyperglycemia) is likely to trigger both. Oxidative stress due to acutely induced hyperglycemia resulted in increased urine GST expression in a mouse model [31]. Recurrence of such events could in time lead to enzymatic exhaustion. Diabetic subjects with decreased π-GST activity are less likely to cope with oxidative stress and, therefore, could more easily develop generalized vascular injury and endothelial dysfunction [32].

Tamm-Horsfall protein appears to rise with increasing diabetes duration, whereas urine albumin excretion, a more conventional marker for the development of nephropathy, remains normal [33]. This may explain why in this study THP was not associated with diabetic, inflammatory or vascular markers.

*Study limitations*: π GST was measured in urine, not in the blood or the cytosolic of renal cells, making thus difficult the interpretation of the precise meaning of our findings. This issue needs to be addressed in future studies.

## Conclusions

In conclusion, the findings suggest that, in young patients with type1 diabetes, lower levels of π-GST:crea ratio correlate with changes in arterial structure and microvascular function. Future prospective studies are warranted to assess whether urine levels of π-GST could be used as biomarker for kidney disease in young patients with type 1 diabetes, and to clarify the above associations.

## Competing interests

The authors declare that they have no competing interests.

## Authors’ contributions

PH designed the renal part of the study, carried out statistical analysis, and drafted the manuscript. PL designed the vascular part of the study, contributed to statistical analysis, and revised the manuscript. Both authors read and approved the final manuscript.

## Pre-publication history

The pre-publication history for this paper can be accessed here:

http://www.biomedcentral.com/1471-2261/14/39/prepub

## References

[B1] Odermarsky M Andersson S Pesonen E Ylä-Hertuala S Liuba P Respiratory infection recurrence and passive smoking in early atherosclerosis in children and adolescents with type1 diabetes Eur J Clin Invest 2008 38 381 388 18445042 10.1111/j.1365-2362.2008.01952.x

[B2] Krantz JS Mack WJ Hodis HN Liu CH Kaufman FR Early onset of subclinical atherosclerosis in young persons with type1 diabetes J Pediatr 2004 145 452 457 15480366 10.1016/j.jpeds.2004.06.042

[B3] Blacher J Pannier B Guerin AP Marchais SJ Safar ME London GM Carotid arterial stiffness as a predictor of cardiovascular and all-cause mortality in end stage renal disease Hypertension 1998 32 570 574 9740628 10.1161/01.hyp.32.3.570

[B4] Järvisalo MJ Raitakari M Toikka JO Putto-Laurila A Rontu R Laine S Lehtimäki T Rönnemaa T Viikari J Raitakari OT Endothelial dysfunction and increased arterial intima-media thickness in children with type1 diabetes Circulation 2004 109 1750 1755 15023875 10.1161/01.CIR.0000124725.46165.2C

[B5] Sweitzer NK LeCaire T Stein JH Keles S Palta M Mitchell GF Increases in central aortic impedance precede alterations in arterial stiffness measures in type 1 Diabetes Mellitus Diabetes Care 2007 30 2886 2891 17686834 10.2337/dc07-0191

[B6] Creager MA Lüscher TF Consentius F Beckman JA Diabetes and vascular disease: pathophysiology, clinical consequences, and medical therapy Circulation 2003 108 1527 1532 14504252 10.1161/01.CIR.0000091257.27563.32

[B7] Cawood TJ Bashir M Brady J Murray B Murray PT O’Shea D Urinary Collagen IV and π-GST: Potential Biomarkers for Detecting Localized Kidney Injury in Diabetes-A Pilot Study Am J Nephrol 2010 32 219 225 20664197 10.1159/000317531

[B8] Holmquist P Torffvit O Jørgensen PE Tørring N Nexø E Sjöblad S Early urinary changes in Tamm-Horsfall protein and epidermal growth factor in diabetic children Pediatric Nephrol 2001 16 488 492 10.1007/s004670100587 11420912

[B9] Harrison DJ Kharbanda R Scott Cunningham D Lellan M Hayes JD Distribution of glutathione S-transferase isoenzymes in human kidney: basis for possible markers of renal injury J Clin Pathol 1989 42 624 628 2738168 10.1136/jcp.42.6.624 PMC1141991

[B10] Conklin DJ Haberzettl P Prough RA Bhatnagar A Glutathione-S-transferase P protects against endothelial dysfunction induced by exposure to tobacco smoke Am J Physiol Heart Circ Physiol 2009 296 1586 1597 10.1152/ajpheart.00867.2008 PMC2685347 19270193

[B11] Holmquist P Ole T Tubular function in diabetic children assessed by urine Glutathion S-Transferase Ped Nephrol 2008 23 1079 1083 10.1007/s00467-008-0770-9 18351395

[B12] Eijkenboom J Van Eijk L Pickkers P Peters W Wetzels J van der Hoeven H Small increases in the urine excretion of glutathionine S-transferase A1 and P1 after cardiac surgery are not associated with chlinically relevant renal injury Intensive Care Med 2005 31 664 667 15812628 10.1007/s00134-005-2608-2

[B13] Branten AJ Mulder TP Peters WH Assmann KJ Wetzels JF Urinary excretion of glutathione S transferases alpha and pi in patients with proteinuria: reflection of the site of tubular injury Nephron 2000 85 120 126 10867517 10.1159/000045644

[B14] Torffvit O Melander O Hulten L Urinary excretion rate of Tamm-Horsfal protein is related to salt intake in humans Nephron Physiol 2004 97 31 36 10.1159/000077600 15153749

[B15] Sejdiu I Torffvit O Decreased urinary concentration of Tamm-Horsfall protein is associated with development of renal failure and cardiovascular death within 20 years in type 1 but not in type 2 diabetic patients Scand J Urol Nephrol 2008 42 168 174 17907053 10.1080/00365590701644691

[B16] Holmquist P Liuba P Urine α-Glutathione S-Transferase, systemic Inflammation and arterial function in juvenile Type1 Diabetes J Diab Compl 2012 26 199 204 10.1016/j.jdiacomp.2012.03.023 22534514

[B17] Torffvit O Agardh CD Kjellsson B Wieslander J Tubular secretion of Tamm-Horsfall protein in type1 (insulin-dependent) diabetes mellitus using a simplified enzyme linked immunoassay Clin Chim Acta 1992 205 31 41 1521339 10.1016/0009-8981(92)90351-p

[B18] Torffvit O Wieslander J A simplified enzyme-linked immunosorbent assay for urinary albumin Scand J Clin Lab Invest 1986 46 545 548 3775240 10.3109/00365518609083711

[B19] Kyhse-Andersen J Schmidt C Nordin G Andersson B Nilsson-Ehle P Lindström V Grubb A Serum cystatin-C, determined by a rapid, automated particle-enhanced turbidimetric method, is a better marker than serum creatinine for glomerular filtration rate Clin Chem 1994 40 1921 1926 7923773

[B20] Odermarsky M Lernmark A Truedsson L Liuba P Cutaneous microvascular dysfunction is associated with human leukocyte antigen-DQ in youths with type 1 diabetes Pediatr Res 2008 63 420 422 18356750 10.1203/PDR.0b013e318165bfd4

[B21] Grubb A Nyman U Björk J Lindström V Rippe B Sterner G Christensson A Simple Cystatin- C based prediction equations for glomerular filtration rate compared with the modification of diet in renal disease prediction equation for adults and the Schwartz and Counahan-Barratt prediction equations for children Clin Chem 2005 51 1420 1431 15961546 10.1373/clinchem.2005.051557

[B22] Krishnan S Fields DA Copeland KC Blackett PR Anderson MP Gardner AW Sex differences in cardiovascular disease risk in adolescents with type1 diabetes Gend Med 2012 9 251 258 22795492 10.1016/j.genm.2012.05.003 PMC3481996

[B23] Schwab KO Doerfer J Hallermann K Krebs A Schorb E Krebs K Winkler K Marked smoking-associated increase of cardiovascular risk in childhood type 1 diabetes Int J Adolesc Med Health 2008 20 3 285 292 19097567 10.1515/ijamh.2008.20.3.285

[B24] Raes A Matthys D Donckerwolcke R Craen M Van Aken S Vande Walle J Renal functional changes in relation to hemodynamic parameters during exercise test in normoalbuminuric insulin-dependent children Acta Paediatr 2007 96 4 548 551 17306011 10.1111/j.1651-2227.2006.00157.x

[B25] Theilade S Lajer M Persson F Joergensen C Rossing P Arterial stiffness isassociated with cardiovascular, renal, retinal, and autonomic disease in type1 diabetes Diabetes Care 2013 36 3 715 721 23193205 10.2337/dc12-0850 PMC3579374

[B26] Heimhalt-El Hamriti M Schreiver C Noerenberg A Scheffler J Jacoby U Haffner D Fischer DC Impaired skin microcirculation in paediatric patients with type1 diabetes mellitus Cardiovasc Diabetol 2013 12 115 23937662 10.1186/1475-2840-12-115 PMC3751195

[B27] Taal M WSigrist MK Fakis A Fluck RJ McIntyre CW Markers of arterial stiffness are risk factors for progression to end-stage renal disease among patients with chronic kidney disease stages 4 and 5 Nephron Clin Pract 2007 107 177 181 10.1159/000110678 17975325

[B28] Ren Y Garvin JL Liu R Carretero OA Cross-talk between arterioles and tubules in the kidney Pediatr Nephrol 2009 24 31 35 18488254 10.1007/s00467-008-0852-8 PMC2697568

[B29] Theilade S Lajer M Jorsal A Tarnow L Parving HH Rossing P Arterial stiffness and endothelial dysfunction independently and synergistically predict cardiovascular and renal outcome in patients with type 1 diabetes Diabet Med 2012 29 990 994 22414297 10.1111/j.1464-5491.2012.03633.x

[B30] Picardi A, valorani MG, Vespasiani Gentilucci U, Manfrini S, Ciofini O, Cappa M, Guglielmi C, Pozzilli P, IMDIAB Group Raised C-reactive protein levels in patients with recent onset type1 diabetes Diabetes Metab Res Rev 2007 23 211 214 16832831 10.1002/dmrr.671

[B31] Fujita H Haseyama T Kayo T Nozaki J Wada Y Ito S Koizumi A Increased expression of Glutathione S-Transferase in renal proximal tubules in the early stages of diabetes: A study of type-2 diabetes in the akita mouse model Exp Nephrol 2001 9 380 386 11701997 10.1159/000052636

[B32] Bin Z Hanchao S Junfu Z Feili L Ying H Effects of simvastin on oxidative stress in streptozotocin-induced diabetic rats: A role for glomeruli protection Nephron Exp Nephrol 2005 101 1 8 10.1159/000085712 15886498

[B33] Torffvit O Sejdiu I Early distal tubular dysfunction is prognostic for development of renal insufficiency within 20 years in type 1 but not in type 2 diabetic patients. Abstract of the 19^th^ world diabetes congress Cape Town, South Africa Diabet Med 2006 23 suppl 4 102 103

